# Assessment of Sleep in Children with Mucopolysaccharidosis Type III

**DOI:** 10.1371/journal.pone.0084128

**Published:** 2014-02-04

**Authors:** Louise Victoria Mahon, Michelle Lomax, Sheena Grant, Elaine Cross, Dougal Julian Hare, James Ed Wraith, Simon Jones, Brian Bigger, Kia Langford-Smith, Maria Canal

**Affiliations:** 1 Division of Clinical Psychology, University of Manchester, Manchester, United Kingdom; 2 Genetic Medicine, St. Mary’s Hospital, Manchester, United Kingdom; 3 Faculty of Medical and Human Sciences, University of Manchester, Manchester, United Kingdom; 4 Faculty of Life Sciences, University of Manchester, Manchester, United Kingdom; Nathan Kline Institute and New York University School of Medicine, United States of America

## Abstract

Sleep disturbances are prevalent in mucopolysaccharidosis Type III (MPS III), yet there is a lack of objective, ecologically valid evidence detailing sleep quantity, quality or circadian system. Eight children with MPS III and eight age-matched typically developing children wore an actigraph for 7–10 days/nights. Saliva samples were collected at three time-points on two separate days, to permit analysis of endogenous melatonin levels. Parents completed a sleep questionnaire and a daily sleep diary. Actigraphic data revealed that children with MPS III had significantly longer sleep onset latencies and greater daytime sleep compared to controls, but night-time sleep duration did not differ between groups. In the MPS III group, sleep efficiency declined, and sleep onset latency increased, with age. Questionnaire responses showed that MPS III patients had significantly more sleep difficulties in all domains compared to controls. Melatonin concentrations showed an alteration in the circadian system in MPS III, which suggests that treatment for sleep problems should attempt to synchronise the sleep-wake cycle to a more regular pattern. Actigraphy was tolerated by children and this monitoring device can be recommended as a measure of treatment success in research and clinical practice.

## Introduction

Mucopolysaccharidosis type III (MPS III/Sanfilippo syndrome) is an inherited metabolic disorder characterised by an absence/defect of lysosomal enzymes needed to break down glycosaminoglycans (GAGs). Accumulation of GAGs leads to progressive dysfunction of cells, tissues and organs [1], [2]. Incidence varies across countries with 1.21 per 100,000 babies affected in the UK [1]. MPS III has three phases [3], with the first phase (1–4 years) characterised by developmental delay, the second phase (4–10 years) by behavioural disturbance, including sleep difficulties, aggressive or destructive behaviours, hyperactivity and attention difficulties, and the third/end phase (10+ years) by progressive loss of skills (especially language), seizures, and problems with mobility and swallowing. The four sub-types of MPS III A-D correspond to variations in enzyme deficiency, with A and B being the most common and D the most rare. There is little clinical difference between subtypes [3], but A might show a more severe course [4] and C a more attenuated course [5].

Sleep difficulties are prevalent in MPS III, particularly settling problems, night-time waking and early morning waking [6]. Both pharmacological and behavioural approaches have been used and although some improvement has been reported, most treatment has been unsuccessful. To date, there are a small number of papers on sleep in MPS III. In four studies using parental questionnaires, between 67–91.5% of MPS III patients exhibited sleep disturbance [7]–[10], including settling difficulties, night-time waking, staying awake all night, crying out, wandering around the house at night, getting into parents’ bed, laughing and singing at night [7], [8]. Colville et al. [8] reported that children with MPS IIIB showed higher rates of early morning waking and night waking compared to sub-type A. The average age of onset of sleep problems was 3.8 years and caregivers noted an association between night-time sleep disturbance and daytime sleepiness or aggressive behaviours [10]. Although no medications were unanimously judged to be effective, melatonin was viewed as the most helpful [10] and 50% of behavioural interventions resulted in meaningful improvements [10], [8]. Clinicians reported 80–95% of MPS III patients experienced sleep difficulties and daytime behaviours were worse when sleep difficulties were more severe, however some reported that behaviour actually improved when sleep was disturbed [11].

Objective measurement of sleep can be achieved by the use of polysomnography (PSG) and actigraphy. Mariotti et al. [12] utilised PSG with six individuals with MPS IIIA aged 7–20 years (mean 14.1 years). Compared to age- and sex-matched controls, MPS III patients displayed less nocturnal sleep (240.5 min vs. 458.3 min), REM sleep (8.03% vs. 21.53%) and slow wave sleep (10.18% vs. 24.63%), but greater daytime sleep (88.8 min vs. 24.8 min). The youngest two patients (aged 7 & 9 years) slept mainly at night but displayed nocturnal waking, whereas children aged 12+ years showed very fragmented sleep of variable duration across night and day. Actigraphy detects body movement to distinguish between wakefulness and sleep. Actigraphy is more precise than sleep diaries [13], correlates highly with PSG [14], is less expensive and less intrusive than PSG, and can be used to gather with naturalistic sleep data over a long period.

It is proposed that there are abnormalities in the circadian rhythm of melatonin concentration in specific developmental disorders, for example an inversion of the circadian rhythm of melatonin has been demonstrated in Smith-Magenis syndrome [15], [16]. Given the presence of irregularly distributed sleep, which appears to be dispersed over day and night in individuals with MPS III, it has been suggested that there is an abnormality of the circadian rhythm of melatonin in this disorder. Guerrero, Pozo, Diaz-Rodriguez, Martinez-Cruz, and Vela-Campos [17] compared twelve patients with MPS III and nine controls aged 6–14 years over a 24-hour period. Compared to the control group, MPS III patients had lower levels of melatonin at night and higher levels during the day. In a mouse model study [18], MPS IIIB mice had higher levels of activity during the resting phase, a reduced resting phase and lower amplitude rhythm compared to age-matched control mice. These findings support the concept of circadian system dysfunction in MPS III, which could in turn act as an important biomarker and clinical outcome measure [19], [20].

The primary aim of the present study was to gather objective, ecologically valid information on sleep and circadian rhythms in children with MPS III. It was hypothesised that MPS III patients would show greater settling difficulties, reduced night-time sleep, poorer quality sleep, increased daytime sleep and an alteration of the circadian system, compared to controls. A secondary aim was to determine the suitability of actigraphy with this patient group and the potential use of actigraphy as a clinical outcome measure. No previous investigations have used actigraphy or salivary melatonin analyses in MPS III and none have combined sleep questionnaires with actigraphy and melatonin testing to examine sleep and circadian alterations in MPS III patients.

## Methods

### Ethics Statement

National Research Ethics Service: North West 12 Research Ethics Committee, Lancaster (REC reference number: 11/NW/0068) approved the study.

### Participants

Eight children with MPS III (5 males, 3 females; mean age 9 years 3 months, SD 4.86, age range 2–15 years) were enrolled through their physicians at the Willink Biochemical Genetics Unit or through the MPS Society UK. Children were selected based on their diagnosis rather than the presence of sleep disturbance, and diagnosis was confirmed by analysis of urine GAGs and specific enzyme analysis. Patients who were involved in a drug study, had a bone marrow transplant, a serious disease affecting another organ, or were near the end of life as advised by their doctor were excluded from this study. Demographic details are displayed in Table 1. Two MPS III participants had epilepsy, and parents of one of these children noted that a lack of sleep at night triggered seizures. The younger patients were not taking any medications, whereas the older patients were prescribed drugs for sleep (e.g. melatonin, chloral hydrate, zopiclone), epilepsy (e.g. sodium valproate), and other symptoms, such as pain. Only one patient was currently prescribed melatonin, but as exogenous melatonin impacts circadian rhythms [21], it was ceased two weeks prior to data collection. This ensured that no lingering effects of melatonin masked the circadian behaviour or the physiologic levels of melatonin in saliva samples. No other medications were altered. A group of age-matched controls (4 males, 4 females; mean age 8 years 7 months, SD 4.85, age range 3–15 years) were selected who did not have a developmental disability, psychiatric disorder, neurological disorder/brain injury or sleep disorder and none were taking medication.

**Table 1:** MPS III Participant Information.

Sex	Age	Ethnicity	MPS III subtype	Current intervention(effectiveness^a^)	Previous treatmentfor sleep problems(effectiveness^a^)
Male	2	Pakistani	B	None	None
Male	4	White Polish	A	None	None
Male	5	White British	B	None	Behavioural advice(parents alreadyused the techniques)
Male	10	Pakistani	B	Melatonin^b^ (good for settling),Loperamide	Herbal medicine(not effective afterthe first week)
Female	10	Pakistani	B	Walking (seems to help)Risperidone (not very helpful)	None
Female	11	White British	A	Gonapeptyl	None
Male	14	White British	A	Chloral hydrate, Zopiclone(both effective in the short-term,not long-term), Levetiracetam,Ibuprofen, Hyoscine	Melatonin (effectivein the short-term, not long-term), Behaviouralmodification (not effective)
Female	15	White British	A	Zopiclone, Clonazepam,Midazolam, Sodium Valproate,Senokot, Movicol, Omeprazole,Glycopyrrolate, Morphine, Paracetamol	Melatonin (no effect),Temazepam (effectiveinitially but became tearful & distressed)

a Effectiveness of treatment as evaluated by parents.

b Melatonin was withdrawn for the study.

Oral and written informed consent was obtained from a parent of each child. Typically developing children aged six to thirteen years also gave their assent (oral and written), and those aged fourteen to fifteen years provided consent (oral and written). All children received a gift voucher for taking part.

### Materials and Procedure

A letter outlining the project was sent to all families with a child with MPS III who were under the care of the Willink Unit or known to the MPS Society UK. Children of colleagues at the University of Manchester or a local NHS Trust formed the control group. Participant information sheets were provided to families who expressed an interest in taking part. A researcher met with each family and the child’s demographic details were gathered. Parents completed the Children’s Sleep Habits Rating Scale (E. Shapiro, personal communication, September 6, 2010), a checklist adapted from the Children’s Sleep Habits Questionnaire [22] to assess sleep problems in patients with MPS III. The checklist required parents to indicate how often each sleep behaviour occurred in a typical week. Items were rated on a three-point scale: ‘usually’ for behaviours occurring five to seven times per week, ‘sometimes’ for those happening two to four times per week, and ‘rarely/never’ for behaviours shown up to once per week.

All children wore an actigraph (Respironics Actiwatch 2/Cambridge Neurotechnology AW4) on their non-dominant wrist for seven to ten days and nights, whilst carrying out their usual activities. A 15 second sampling interval was employed. Previous research found that a minimum of five nights data are needed to provide meaningful actigraphic recordings of a child’s sleep, and actigraphic monitoring should cover at least seven nights to compensate for factors such as illness, technical problems and non-compliance [23]. Recording for a minimum of a week meant that data could be gathered over school days and over the weekend, as well as allowing for potential loss of data. The sleep parameters of interest were sleep onset latency (time between ‘lights out’ and sleep onset), total night-time sleep duration, sleep efficiency (percentage of time asleep between time of ‘lights out’ and ‘get up’), wake after sleep onset (WASO, total number of minutes awake between sleep onset and time of final waking), time in bed (minutes) and total daytime sleep duration (total number of minutes asleep between time of final waking and bed time). To avoid confounding effects of large seasonal variation in circadian rhythms, data was gathered within a six-month period and during school term time to ensure comparability across subjects. Caregivers noted their child’s bed times, get up times, lights on/off times and any night-time events in a daily diary.

To allow examination of endogenous melatonin levels, parents took saliva samples from their child at three time points (between 6–8 h, 10–12 h, & 22–24 h), on the first and last day of actigraphic recording. Samples were collected using a suction catheter (de Lee suction catheter; Argyle, Sherwood Medical, Tullamore, Ireland) and were frozen at −20°C until analysis. Night collection was performed under dim light conditions. Samples were collected in accordance with procedures outlined by the enzyme-linked immunosorbent assays (ELISA) kit manufacturer (www.IBL-International.com), to allow testing using Non-Extraction Melatonin Saliva ELISA (IBL, Hamburg, Germany). Preliminary analyses indicated that the data were reliable as standard deviations were less than 20% of the mean. One outlier was removed. Some saliva samples were missing or incomplete and were excluded (pairwise), but 88.5% of saliva samples were useable.

### Analysis

Actigraphic data were transferred to Actiware version 5.5 software (Respironics). Rest intervals were set based on diary information and review of the actogram (where activity and light intensity decreased). Actigraphic and questionnaire data were entered onto SPSS version 19 for analysis. One night’s actigraphy data had to be excluded from the analysis for several participants due to illness or non-compliance, but a minimum of seven nights’ data were available for all subjects. An alpha level of 0.05 was used for all statistical tests.

## Results

### Children’s Sleep Habits Rating Scale

The average age of onset of sleep problems for MPS III patients was 2 years (SD 2.33, range birth-7 years). Sleep problems identified by parents included difficulties settling, waking up during the night, early morning wakening, and sleeping too little. Most children (62.5%) needed a parent in the room whilst trying to sleep. Half of the children fell asleep within twenty minutes on some nights, but 37.5% rarely or never did. Most parents believed their child slept too little on at least two nights each week, but on other nights they seemed to sleep the right amount. The majority of children woke up once (62.5%), or multiple times (87.5%) at night. Most of the children (75%) displayed disruptive behaviour at night (e.g. screaming, singing, laughing), and 25% of children displayed dangerous behaviours (e.g. running outside, playing with appliances). All children were restless and moved a lot at night and 50% of children slept during the day.

Mann Whitney U tests demonstrated that the MPS III group had significantly more disturbed sleep in all areas assessed by the Children’s Sleep Habits Rating Scale. Results are displayed in Table 2.

**Table 2:** Comparison of MPS III and Control Group Data on the Children’s Sleep Habits Rating Scale.

	MPS III		Controls					
	(n = 8)		(n = 8)					
Subscale	Mean	Median	Mean	Median	*U*	*z*	*r*	*p*
	(SD)	(IQR)	(SD)	(IQR)				
Bedtime resistance	9.5 (2.1)	9.5 (5)	6.3 (0.5)	6.0 (1)	62.0	−3.3	0.8	0.001**
Sleep onset delay	2.3 (0.7)	2.0 (1)	1.1 (0.4)	1.0 (0)	57.5	−2.9	0.7	0.006**
Sleep duration	6.1 (1.6)	6.0 (1)	3.0 (0)	3.0 (0)	60.0	−3.3	0.8	0.001**
Sleep anxiety	6.4 (2.7)	5.5 (4)	4.0 (0)	4.0 (0)	56.0	−2.9	0.7	0.007**
Night wakings	5.9 (1.5)	6.5 (2)	3.9 (1)	3.5 (2)	55.0	−2.5	0.6	0.016*
Night behaviours	3.3 (1.3)	3.0 (2)	2.0 (0)	2.0 (0)	56.0	−2.9	0.7	0.007**
Parasomnias	9.8 (1.7)	9.0 (4)	6.4 (0.7)	6.0 (1)	63.0	−3.3	0.8	0.000**
Sleep disordered breathing	4.8 (1.8)	4.0 (3)	3.0 (0)	3.0 (0)	56.0	−2.9	0.7	0.007**
Daytime sleepiness	16.0 (4.1)	14.5 (8)	12.3 (2.1)	12.5 (2)	51.0	−2.0	0.5	0.045*

SD, standard deviation; IQR, interquartile range. Items were grouped into nine subscales. Each item was assigned a score between 1 and 3 and some items were reverse-scored to ensure that a higher score represented poorer sleep.

* *p*<.05, **p<.01.

### Actigraphy

As suggested by Acebo et al. [23], actigraphic data for each participant were averaged over the recording period prior to analysis. Actigraphic data for each MPS III patient can be seen in Table 3. Night-time sleep is diminished in some patients, particularly P8 who only slept for an average of 190 minutes per night, however most children slept a normal amount at night. Five out of eight children slept during the day on occasions during actigraphic monitoring, but most children did not nap every day. Sleep onset latency was markedly high in the majority of children. One of the youngest children settled very quickly, within approximately 6 minutes, but the eldest two children had distinctly long sleep onset latencies of approximately 1 hour 10 minutes and 3 hours respectively. The remaining five children took between 25 minutes and 40 minutes to enter a sleep state. Sleep efficiency was diminished in some children, with the eldest child sleeping only 28% of the time in bed and the five year old sleeping just 59% of the time in bed. WASO was elevated in all children, notably the oldest two subjects, and it ranged from 64 minutes to 207 minutes per night across participants.

**Table 3:** Sleep Parameters (per night) for MPS III Participants Averaged over the Recording Period.

	Age (years)								
Sleep variable	P1	P2	P3	P4	P5	P6	P7	P8	Control
	2	4	5	10	10	11	14	15	mean
Time in bed (min)	570.9	589.5	593.8	579.1	528.8	558.2	659.4	397.4	544.9
Night-time sleep (min)	507.2	504.5	396.6	497.3	464.9	484.0	532.8	190.2	479.0
Daytime sleep (min)	46.5	0	1.2	0	40.6	0	16.4	6.0	1.7
Sleep onset latency (min)	38.6	6.3	24.8	30.4	32.2	39.1	70.9	183.3	14.2
Sleep efficiency (%)	81.0	79.6	59.3	79.2	73.7	77.5	70.5	28.6	80.8
WASO (min)	63.7	85.0	197.2	81.8	63.9	74.2	126.6	207.2	75.1

WASO, wake after sleep onset.

**Figure 1:**
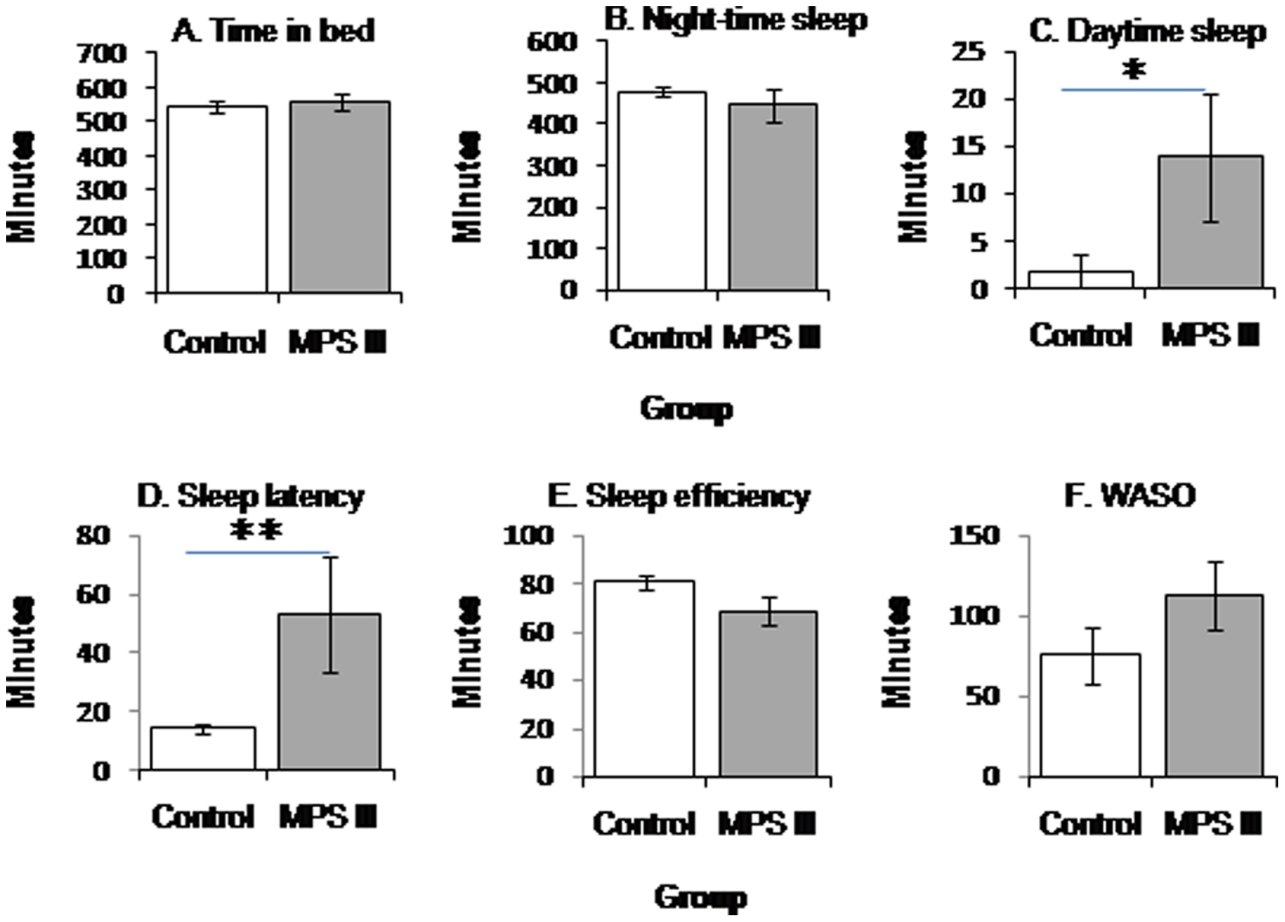
Mean actigraphic results of MPS III (*n = *8) and control groups (*n = *8). Error bars represent standard error of the mean. (A) Duration of time in bed. (B) Duration of night-time sleep. (C) Duration of daytime sleep. (D) Sleep onset latency. (E) Sleep efficiency. (F) Wake after sleep onset. * p<0.05, ** p<0.01.

Actigraphic data from the groups were analysed using Mann-Whitney U tests and results are displayed in Table 4. Sleep onset latency was significantly longer in children with MPS III compared to the control group. Night-time sleep duration and time in bed did not differ significantly between the groups. WASO was higher in the MPS III group, but it was not significantly different from the control group. Although sleep efficiency was lower in the patient group compared to controls, it did not reach statistical significance. Daytime sleep duration was significantly longer in MPS III participants than controls. Group means can be seen in Figure 1.

**Table 4:** Comparison of Sleep Parameters in Children with MPS III and Controls.

	MPS III	Controls				
	n = 8	n = 8				
Sleepparameter	Median(IQR)	Median(IQR)	*U*	*z*	*r*	*p*
Time inbed (min)	575.0(56.6)	531.6(55.6)	44.0	−1.3	0.3	0.23
Night-timesleep (min)	490.6(92.9)	496.2(61.4)	32.0	0	0	1.0
Daytimesleep (min)	3.6(34.6)	0(0)	48.5	−2.0	0.5	0.046*
Sleep onsetlatency (min)	35.4(36.8)	14.9(7.6)	56.0	−2.5	0.6	0.01**
Sleepefficiency (%)	75.6(17.4)	82.8(15.1)	17.5	−1.5	0.4	1.4
WASO(min)	83.4(113.1)	60.4(94.7)	43.0	−1.2	0.3	0.279

WASO, wake after sleep onset; SD, standard deviation.

* *p*<.05, **p<.01.

In the MPS III group, Spearman’s rank correlation coefficients revealed a positive correlation between age and sleep onset latency, *r_s_* = 0.755, *n = *8, *p = *0.031, and a negative correlation between age and sleep efficiency, *r_s_* = −0.719, *n = *8, *p = *0.045. Age was not significantly correlated with any other variables.

### Melatonin Analyses

**Figure 2:**
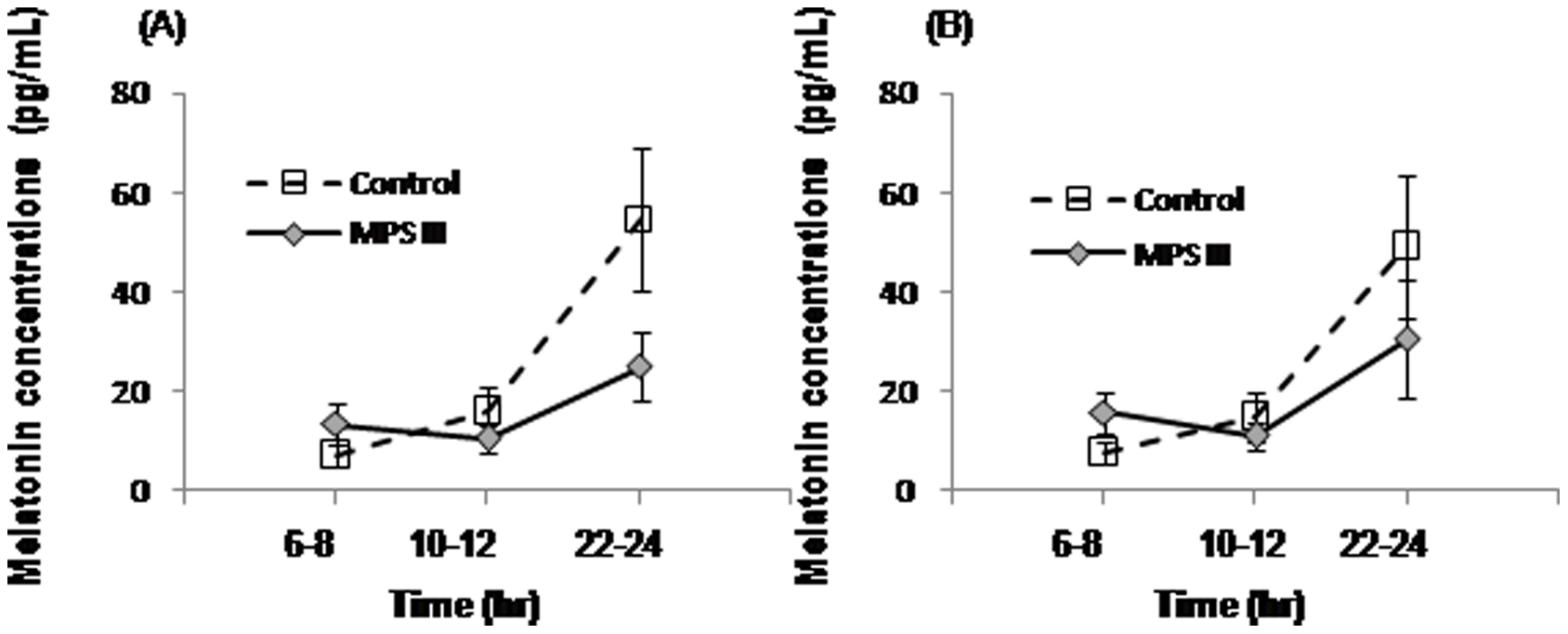
Melatonin concentrations (average ± SEM) in MPS III patients and controls. Saliva samples were collected at the times shown on. (A) First day of actigraphic monitoring. (B) Last day of actigraphic recording.

Melatonin levels by day and time of collection are shown in Table 5 and the groups are compared in Figure 2. To determine whether melatonin concentrations were influenced by day of collection (first day vs. last day), Wilcoxon signed-rank tests were conducted on patient and control group data, which revealed no significant effects of day (*p*>0.05). A Friedman test indicated that there was a statistically significant difference in melatonin concentrations for the control group across time points (Time 1∶6–8 h, Time 2∶10 h-12 h, Time 3∶22–24 h) on the first day, χ^2^(2) = 10.33, *p = *0.002, and last day, χ^2^(2) = 9.33, *p = *0.006. Post hoc Wilcoxon tests with Bonferroni correction (adjusted alpha level of 0.016) showed a significant difference between Time 2 and Time 3 on both days (*p* = 0.016, *r = *0.61), but the difference between Time 1 and Time 3 was just above significance (*p = *0.031, *r* = 0.59). There were no reliable differences across time points for the MPS III group on the first day χ^2^(2) = 0.50, *p = *0.931, or last day χ^2^(2) = 2.80, *p = *0.367. Visual inspection of the data suggested that the MPS III group had higher melatonin concentrations at 6–8 h and lower levels at 22–24 h, compared to controls, however Mann-Whitney tests found no significant differences between groups.

**Table 5:** Median (IQR) Melatonin Concentrations across Groups, Time points and Days.

	Melatonin concentrations (pg/mL) by day and time					
	First day			Last day		
Group	6–8 h	10–12 h	22–24 h	6–8 h	10–12 h	22–24 h
MPS III	14 (14.1)	6.6 (8.6)	19 (20.4)	11 (10)	11.1 (10.2)	38.2 (40)
Controls	4.3 (10.8)	11.1 (29)	51.5 (87)	7.7 (9)	9 (27.8)	45.7 (87.7)

IQR, interquartile range.

## Discussion

Children with MPS III took longer to fall asleep and slept more during the day compared to age-matched typically developing children, thus supporting the initial hypotheses. There were trends towards diminished sleep efficiency and increased nocturnal wakefulness in the patient group. Night-time sleep duration was not depleted in the patient group which contradicted the hypothesis. As predicted, responses on the Children’s Sleep Habits Rating Scale revealed that parents of children with MPS III reported greater sleep disturbance in all domains, compared to parents of typically developing children. In the patient group, increased age was associated with greater sleep onset latency and lower sleep efficiency. As hypothesised, MPS III children showed disruption in their circadian system.

The MPS III group took an average of 53 minutes to fall asleep compared to the expected 20–30 minutes [24], [25]. The prolonged sleep onset latency in MPS III suggests difficulties initiating sleep that could relate to low night-time melatonin levels. On average, MPS III patients slept during the day for 14 minutes, with those in the late stage sleeping more during the day than those in the middle phase. This was in line with previous PSG findings [12]. It should be noted that daytime sleepiness in children is associated with behavioural problems, depression, and reduced quality of life [26]. With increasing age, children with MPS III took longer to fall asleep and their sleep was less efficient. Children normally sleep for at least 80% of their time in bed [27], [28], but the average of the MPS III group was 68.7%. However sleep efficiency for some children was only just below normal with only two participants showing very poor sleep efficiency (59.3% and 28.6% respectively). The length of time that MPS III children were awake at night was very high, with an average of almost two hours per night and such wakefulness was particularly noticeable in the two oldest children.

In Mariotti and colleagues’ [12] paper night-time sleep was reduced in MPS III. In the current study average night-time sleep in the MPS III group was lower than controls, however it was not significantly different. Some patients’ sleep varied from one night to the next, and when sleep was averaged over the recording period, children with MPS III appeared to be getting a sufficient quantity of sleep. All Mariotti and colleagues’ [12] subjects were MPS IIIA, whereas the current study included both A and B subtypes. This might account for some of these differences, as might the disparity in subjects’ ages across the two studies.

Results from the Children’s Sleep Habits Rating Scale showed that in comparison to typically developing children, parents of children with MPS III saw their child as having more difficulties with bedtime resistance, falling asleep, sleep duration, sleep anxiety, night waking, night behaviours, parasomnias, sleep disordered breathing and daytime sleepiness. The average age of onset of sleep difficulties was 2 years old, but sleep disordered breathing (snoring, snorting/gasping and apnea) can be present from birth in some instances [29], [30], [6].

In a mouse model paper [18], comparable results to this study were reported. MPS IIIB mice had higher levels of activity during the resting phase, a reduced resting phase and lower amplitude rhythm. This could explain napping during the active phase, night-time waking and nocturnal activity.

Salivary melatonin analyses showed altered circadian rhythm of melatonin concentration in MPS III with lower melatonin levels at night and higher levels early morning. However these differences were not significantly different from controls, partly due to the small sample size and large variability within the groups. Melatonin levels were differentiated at specific time points over day and night in typically developing children. However in MPS III patients, no reliable differences across time were found. In Canal and colleagues’ [18] study, MPS IIIB mice had a lower sensitivity to light at the behavioural (shorter phase shift after a light pulse in the dark) and the molecular level (decreased expression of vasointestinal polypeptide protein in the suprachiasmatic nucleus (SCN)). If comparable processes hold in humans with MPS III, this could account for the abnormal melatonin levels observed in the current study.

Given the abnormal melatonin concentrations observed in MPS III, exogenous melatonin is likely to be the most effective pharmacological intervention, as substantiated by previous research [11]. The strongest evidence for the efficacy of melatonin is for reducing sleep onset latency, but there is less support for its effect on nocturnal waking [31]. Compared to the usual fast release form, sustained-release melatonin can improve sleep maintenance in children with neurodevelopmental disorders [32], but such treatment could exacerbate daytime sleepiness, which should be avoided in patients who already display tiredness during the day. Moreover, melatonin should be used with caution given concerns about its use with children, its long-term effects are unknown [33] and there is some evidence of an increase in seizures in children with neurodevelopmental disorders with epilepsy [34].

As with much research into very rare disorders, the current sample size was small with limited statistical power to detect between-group differences or disparities between subtypes or stages of the disorder. Inspection of standard deviations revealed the MPS group had more variability on all sleep measures compared with controls, and sleep difficulties, particularly sleep onset latency and sleep efficiency, worsened as the children aged and the disease progressed.

With regard to the feasibility of actigraphy with this population, it is inherently more accurate than diary entries and parents reported few problems with their child tolerating the watch, which suggests it is suitable for wider use in both clinical practice and research with MPS III patients.

Further investigations are needed to validate the findings of this study. With more resources, a larger sample including adults as well as children, would substantiate these results and provide greater clarity of sleep at different stages of the disorder. Some parents of children with MPS III reported that occasionally their child would not sleep at all for one night and this unpredictability makes it difficult to plan their daily lives. Recording over a longer time period of several weeks, would allow more variability across nights to be captured. Rather than averaging data across nights, time series analysis could identify patterns and allow periods of sleeplessness to be predicted and a longitudinal study following MPS III patients through the progression of the disorder would document more precisely how sleep patterns change with age and/or subtypes.

A number of promising treatments for MPS III which target the central nervous system are currently being studied, including enzyme replacement therapy, hematopoietic stem cell transplantation, gene therapy, and substrate reduction therapy [35]. Until a clinically efficacious treatment for MPS III has been developed, it remains for clinicians to target manifestations of the condition, including sleep disturbance, to improve the quality of life of individuals and their families.
